# Multi-modal dataset creation for federated learning with DICOM-structured reports

**DOI:** 10.1007/s11548-025-03327-y

**Published:** 2025-02-03

**Authors:** Malte Tölle, Lukas Burger, Halvar Kelm, Florian André, Peter Bannas, Gerhard Diller, Norbert Frey, Philipp Garthe, Stefan Groß, Anja Hennemuth, Lars Kaderali, Nina Krüger, Andreas Leha, Simon Martin, Alexander Meyer, Eike Nagel, Stefan Orwat, Clemens Scherer, Moritz Seiffert, Jan Moritz Seliger, Stefan Simm, Tim Friede, Tim Seidler, Sandy Engelhardt

**Affiliations:** 1 DZHK (German Centre for Cardiovascular Research), partner site Heidelberg/Mannheim, Heidelberg, Germany; 2 Department of Cardiology, Angiology and Pneumology, Heidelberg University Hospital, Heidelberg, Germany; 3 Informatics for Life Institute, Heidelberg, Germany; 4 DZHK (German Centre for Cardiovascular Research), partner site Hamburg/Kiel/Lübeck, Hamburg, Germany; 5 Department of Diagnostic and Interventional Radiology and Nuclear Medicine, University Medical Center Hamburg-Eppendorf, Hamburg, Germany; 6 Clinic for Cardiology III, University Hospital Münster, Münster, Germany; 7 DZHK (German Centre for Cardiovascular Research), partner site Greifswald, Greifswald, Germany; 8 Institute of Bioinformatics, University Medicine Greifswald, Greifswald, Germany; 9 DZHK (German Centre for Cardiovascular Research), partner site Berlin, Berlin, Germany; 10 Deutsches Herzzentrum der Charité (DHZC), Institute of Computer-assisted Cardiovascular Medicine, Berlin, Germany; 11 Charité - Universitätsmedizin Berlin, Corporate Member of Freie Universität Berlin, Humboldt-Universität zu Berlin, Berlin, Germany; 12 Fraunhofer Institute for Digital Medicine MEVIS, Bremen, Germany; 13 DZHK (German Centre for Cardiovascular Research), partner site Lower Saxony, Göttingen, Germany; 14 Department of Medical Statistics, University Medical Center Göttingen, Göttingen, Germany; 15 DZHK (German Centre for Cardiovascular Research), partner site RhineMain, Frankfurt, Germany; 16 Institute for Experimental and Translational Cardiovascular Imaging, Goethe University, Frankfurt am Main, Germany; 17 DZHK (German Centre for Cardiovascular Research), partner site Munich, Munich, Germany; 18 Department of Medicine I, LMU University Hospital, LMU Munich, Munich, Germany; 19 Department of Cardiology, University Heart and Vascular Center Hamburg, University Medical Center Hamburg-Eppendorf, Hamburg, Germany; 20 Department of Cardiology, University Medicine Göttingen, Göttingen, Germany; 21 Department of Cardiology, Kerckhoff-Clinic, Campus Kerckhoff of the Justus-Liebig-Universität Gießen, Gießen, Germany

**Keywords:** Structured reports, Multi-modal, Federated learning, DICOM, Transcatheter aortic valve replacement, Data-filtering

## Abstract

**Purpose** Federated training is often challenging on heterogeneous datasets due to divergent data storage options, inconsistent naming schemes, varied annotation procedures, and disparities in label quality. This is particularly evident in the emerging multi-modal learning paradigms, where dataset harmonization including a uniform data representation and filtering options are of paramount importance.

**Methods** DICOM-structured reports enable the standardized linkage of arbitrary information beyond the imaging domain and can be used within Python deep learning pipelines with highdicom. Building on this, we developed an open platform for data integration with interactive filtering capabilities, thereby simplifying the process of creation of patient cohorts over several sites with consistent multi-modal data.

**Results** In this study, we extend our prior work by showing its applicability to more and divergent data types, as well as streamlining datasets for federated training within an established consortium of eight university hospitals in Germany. We prove its concurrent filtering ability by creating harmonized multi-modal datasets across all locations for predicting the outcome after minimally invasive heart valve replacement. The data include imaging and waveform data (i.e., computed tomography images, electrocardiography scans) as well as annotations (i.e., calcification segmentations, and pointsets), and metadata (i.e., prostheses and pacemaker dependency).

**Conclusion** Structured reports bridge the traditional gap between imaging systems and information systems. Utilizing the inherent DICOM reference system arbitrary data types can be queried concurrently to create meaningful cohorts for multi-centric data analysis. The graphical interface as well as example structured report templates are available at https://github.com/Cardio-AI/fl-multi-modal-dataset-creation.

**Supplementary Information:**

The online version contains supplementary material available at 10.1007/s11548-025-03327-y.

## Introduction

Multi-modal deep learning is becoming increasingly prominent in medicine, but large-scale datasets are needed to train modern architectures effectively [16]. The predictive performance of these models improves significantly with the training dataset size. However, such large-scale, publicly available datasets are rare, particularly in the multi-modal medical domain. This scarcity is attributed to strict privacy regulations, such as the General Data Protection Regulation (GDPR)^1^ in Europe, which impose substantial barriers to centralized data storage and sharing.

One renowned method, which can circumvent privacy concerns is federated learning (FL) [17]. FL inverts the common paradigm of central data storage by sending the model to each data-owning institution, where local training is performed, before the model weights are sent back to a central instance for averaging. However, due to the missing possibility of manually inspecting the data at each data-holding institution, data harmonization is of paramount importance to enable a meaningful model training. In multi-modal scenarios this becomes even more complicated, which is evident in the low number of consortia in the field [23]. Still, utilizing information from multiple modalities becomes more relevant, but curating the respective cohorts is challenging, particularly across multiple centers. One approach is to use data formats that enforce predefined templates for structuring the data. Despite the large number of possible templates one can adhere to, data across hospitals is far from being standardized [14].

An interactive dashboard that can be operated without programming knowledge would be beneficial to enable intuitive multi-center cohort creation, e.g., on retrospective data. If the data was integrated in coherent templates within a single database, an intuitive filtering with linked graphical filter options between the different modalities can be realized. One could iteratively refine the filter conditions, i.e., the query to circumvent samples with missing modalities or metadata. In the federated setting, where data is distributed across multiple locations, a central dashboard summarizing all data would be the best solution. Unfortunately, this is hampered by privacy laws. Second best is the possibility to copy the exact query from one location and apply it to the data of another location, but this again is dependent on adherence to the same templates.

**Fig. 1:**
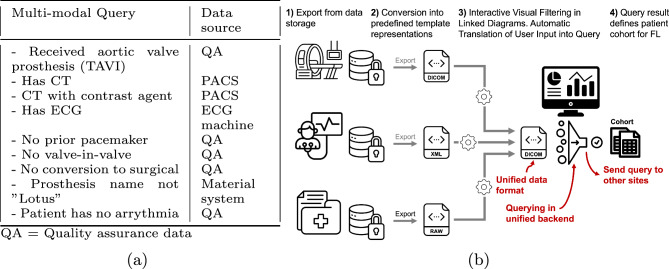
**a** Example multi-modal query with the respective hospital information system the data resides in. **b** Our proposed workflow for unified filtering of data from (**a**). The data is first converted into DICOM using predefined templates. Subsequently, the data is loaded into a research PACS, leveraging the inherent matching capabilities of DICOM, which enables filtering through a graphical user interface

In this study we provide a proof-of-concept that one can make use of the DICOM standards inherent matching capabilities to enable above requirements in imaging fields [19]. DICOM allows for standardized storage of images, waveforms (electrocardiography), segmentations, and metadata in a format termed structured reports (SR) [3, 4]. While other standards such as Fast Healthcare Interoperability Resources (FHIR)^2^ or OpenEHR^3^ exist, leveraging only one data format, i.e., DICOM in imaging-focused scenarios can simplify federated cohort selection. Our approach streamlines the data curation across locations by converting all data to DICOM and uploading into a picture archiving and storage system (PACS) for research. The DICOM standard ensures harmonized data representation since valid objects can only be created if one adheres to the respective templates. Due to standardized representation in a PACS it is possible to create a graphical dashboard that allows for intuitive filtering of the data. The proposed workflow as well as an example query for a hypothetical distributed multi-modal scenario is shown in Fig. 1. To summarize, our work makes the following contributions:

- We introduce a method that allows for harmonization of imaging, waveform, and respective metadata by leveraging the DICOM standard and its inherent matching capabilities.
- We provide publicly available scripts for creation and conversion of data into DICOM especially structured reports. We implement several SR templates in object-oriented Python that enable their usage in deep learning pipelines.
- We introduce an intuitive dashboard that allows for filtering and cohort creation of any DICOM images, waveform, segmentation, and metadata (SRs) residing in a PACS. The filtering can be done iteratively with linked views of the different data types that update instantly.
- We maintain a high level of flexibility in our filtering dashboard by allowing users to customize which attributes to filter and select their preferred graphical representations.
- We show that our created DICOM data ensure interoperability by being able to be queried in standard query language (SQL) as well as ensure possible conversion to other standards such as FHIR.
- The source code is publicly available for re-usage at https://github.com/Cardio-AI/fl-multi-modal-dataset-creation.

A previous version of this article was published at the German Conference on Medical Image Computing [24], where we presented the general feasibility to filter data in a single location training scenario. In this extension we install the federated infrastructure together with our designed filter tool at all locations within our FL consortium. Each location exports the available data from their clinical PACS, pairs it with potentially present labels, converts the data to DICOM representation, and uploads everything into the presented tool. After performing cohort selection at each individual location, the data is exported to subsequently enable a federated training. We generate structured reports from additional templates for a more diverse and larger set of multi-modal medical data and subsequently investigate its filtering capability.

## Interoperability of medical data

Integrating machine learning models into clinical workflows requires interoperability between existing systems, achievable through adherence to established standards. Many organizations have opted to use FHIR because it offers a flexible and adaptable framework that supports a wide range of healthcare data formats and facilitates integration with modern technologies [11, 18]. FHIR has been adopted for its robust interoperability capabilities in projects like the German Medical Informatics Initiative,^4^ where it enables data sharing across diverse healthcare systems [6]. However, introducing another standard would make harmonizing data across locations even more challenging in our use case for medical images, ECGs, and respective metadata. In particular, since DICOM allows for linking images and waveforms with their respective related information, many use cases can be effectively addressed.

**Fig. 2:**
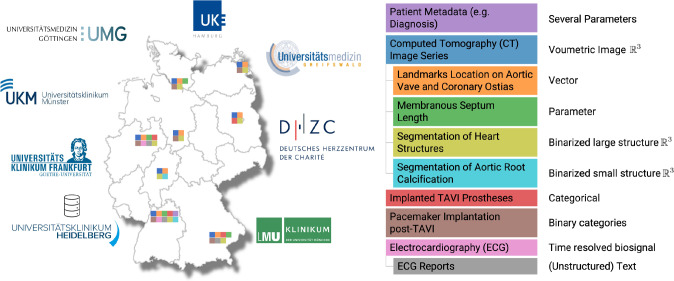
Different data types relevant for prediction of outcome after minimally invasive transcatheter aortic valve implantation (TAVI). The data is heterogeneously distributed across locations in the consortium in terms of type (indicated by the color scheme) and quantity. Derived information for an original data source is indicated by the hierarchy on the right hand side

DICOM-structured reports represent a standardized method for the encoding, transmission, and storage of diagnostic findings. In addition to images, they also allow the linking of other data types such as, e.g., text, audio, time points, or other measurements. Each SR must have a defined template that consists of a sequence of content items (see Fig. 2b in the Appendix). Each content item defines a name-value pair that encodes a domain-specific property or concept. Concept names must follow standard medical ontologies and terminologies such as the Systematized Nomenclature of Medical Clinical Terms (SNOMED CT), which ensures uniqueness and cross-domain interpretability [13]. The value can take up to four different types: it can also be a coded concept, it might include a number, comprise one or more sets of points or other graphic data such as surfaces, or can be made up of plain text (see Fig. 1b in the Appendix).

Using DICOM-structured reports may provide significant benefits such as standardization of imaging and waveform data across different devices and providers, enhanced data accuracy through structured documentation, and improved efficiency in storing and retrieving diagnostic information, which is essential for medical imaging and electrocardiography workflows [3]. Due to the adherence to standardized templates a conversion between DICOM and FHIR or other structured formats is straightforward.

Recently, the Python library highdicom was introduced, which employs an object-oriented approach toward SRs simplifying their handling within deep learning pipelines [1]. However, they have only implemented template identity (TID) 1500 Measurement Report so far, as their primary focus has been on radiology and pathology. The implementation of other templates allows for extension to other fields. Another common data type in radiology is segmentation in which one or multiple structures can be compartmentalized. Each segment is described by a specific label encoded by a coded concept similar to the name of a content item in SRs that ensures uniqueness. Segments can also be referenced in a SR providing another level of entwinement.

## Materials and methods

### Requirements

An application that allows consistent data integration, matching, and cohort selection should fulfil seven requirements.

- **R1 Integration**: Consistent representation of data from different formats in a unifying interface. All required data shall be storable at one place in the same data format without the need to query multiple databases.
- **R2 Unambiguous Matching**: Different data types from the same patient must be filterable together. Concurrently, they must be filterable independent of other types and multiple conditions must be evaluable on the instance level.
- **R3 Exchangeability of Search Query**: The search query from one location should be transferable and applicable to another location in the same way.
- ** R4 Intuitive Cohort Selection**: Graphical visualization and interactive filtering is desirable, while more complicated textual queries should also be possible. The diagrams should be interconnected, ensuring that filtering based on one attribute dynamically updates the other representations known as linked views.
- **R5 Flexibility**: The filtering options should be modifiable depending on the operators needs for which attributes to filter.
- **R6 Conversion**: The data residing in the platform shall be easily convertible between different standards, i.e., in our case between DICOM and, e.g., FHIR.
- ** R7 Independent Platform or Extension to an Existing Platform**: Usage shall be enabled as a stand-alone application as well as an extension to existing frameworks such as Kaapana [21].

### Deployment in a dockerized application

One renowned platform that aims at facilitating the application of algorithms on real world clinical data is Kaapana^5^ [21]. In addition to the possibility of deploying containerized algorithms directly in the platform they also provide a graphical filter tool for cohort selection based on the imaging data in the internal picture archiving and communication system (PACS). However, multi-modal dataset creation is not supported as only image related attributes can be filtered and links to other forms of data (diagnoses, geometric relations, etc.) are not included.

We showcase a possible integration of our developed platform by incorporation into Kaapana. Similar to their inbuilt functionality we also select Opensearch as the tool of choice for enabling graphical cohort filtering.^6^ Due to its deployability in a Docker container our tool can also serve as a stand-alone tool, which can be linked to any PACS. Opensearch’s filtering plots are highly customizable due to the JSON-based representation scheme, which also allows for fast query times with a unified data representation. While we believe our presented layout is a good compromise between complexity and usability, other researchers might want to include further elements or plots and subsequently use them for filtering, which Opensearch allows out of the box.

A patient can have multiple reports or segmentations, which can contain for example different diagnoses or labels for different studies. Filtering these interlinked data types on the same level requires the child element to be aware of its parent due to the possible reference of multiple instances within one object. By default Opensearch can only filter data on the first level, i.e., one series such as a CT scan. To enable the filtering and visualization of **linked data**, which is the main advantage of SRs, we had to use nested queries. With these queries one can filter on two levels, i.e., a parent is evaluated on the level of its child. For performance reasons Opensearch flattens the data in their internal database scheme per attribute That enables a coherent data representation and standardized creation of filtering plots. However, child objects are also flattened and conditions cannot be evaluated on a child level. With nested objects we can evaluate whether multiple conditions are true for one instance, i.e., our children are treated as individual objects. As an example we might want all segmentations that have the left and right ventricle included (**AND** linkage). Since segmentations reference a series on the whole, we would also receive all instances that have only one of both segmented (**OR**). To visualize the results of such nested queries, one cannot use the default visualizations of Opensearch, one must rather use the declarative language Vega to create custom plots.^7^ An illustration of nested queries and their difference to conventional queries in Opensearch is shown by example queries in the Appendix. We provide the query in two formats, Opensearch’s native domain-specific query language (DSL) based on JSON and standard query language (SQL). Opensearch uses a JSON-based query language to enable native web based querying.

## Evaluation

The evaluation presented in this paper is conducted on a specific use case in cardiology with real world clinical data. A federated training is performed on the created cohorts the results of which are presented elsewhere [25].

### Federated learning consortium

**Fig. 3:**
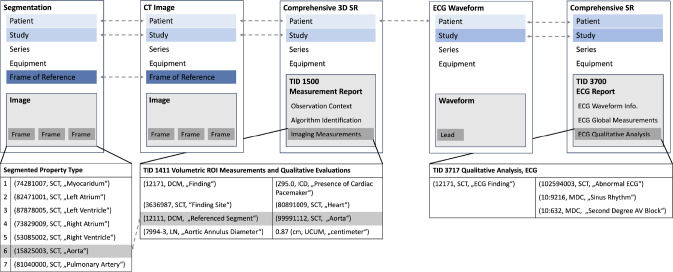
Relevant data and their relationship to predict pacemaker dependency after heart valve replacement. Structured Reports can reference, e.g., images, waveforms, regions, and segments. Similar to highdicom we have also chosen the general Comprehensive (3D) SR due to its high generalizability [1]

Within the German Centre for Cardiovascular Diseases (DZHK)^8^ and further German Hospitals we have established a FL consortium spanning across eight university clinics (see Fig. 2). The use case we want to address is the outcome prediction after minimally invasive transcatheter aortic valve implantation (TAVI) [22]. In the literature multiple possible impact factors on outcome derived from, e.g., computed tomography and electrocardiography scans are reported, such as the length of the membranous septum or the presence of a left bundle branch block (see Fig. 3) [7]. In general, the individual ECG scans can indicate existing disturbed heart stimulus conduction, while CTs provide further information about geometric relations and calcification. Additionally, patient characteristics such as a history of heart disease or diabetes mellitus can yield further insights. However, a dedicated analysis of the interactions and an automated analysis on the raw input data with deep learning across multiple institutions is missing [25].

### Data collection and harmonization

Every institution was required to export the relevant data they possess from their internal clinical information systems. The resulting dataset includes one CT scan, one pre-TAVI ECG, the corresponding quality assurance metadata, and the invoice code whether a pacemaker was implanted post-TAVI. However, the data resides in the different clinical information systems in other formats, which must be harmonized. A concurrent filtering of all data modalities in a single tool that allows for temporal alignment of different procedures is desirable for uniform dataset creation for federated training. In total we used 15 SR templates^9^ to store all parameters per modality (see Tab. 2 in the Appendix). To ensure the validity of our SR templates as DICOM objects, we used dciodvfy from David Clunie.^10^ To verify the intended functionality of our tool we additionally uploaded six public datasets and aimed at filtering out the needed subset for our pacemaker prediction scenario at each location [5, 8, 10, 12, 20, 26].

**Fig. 4:**
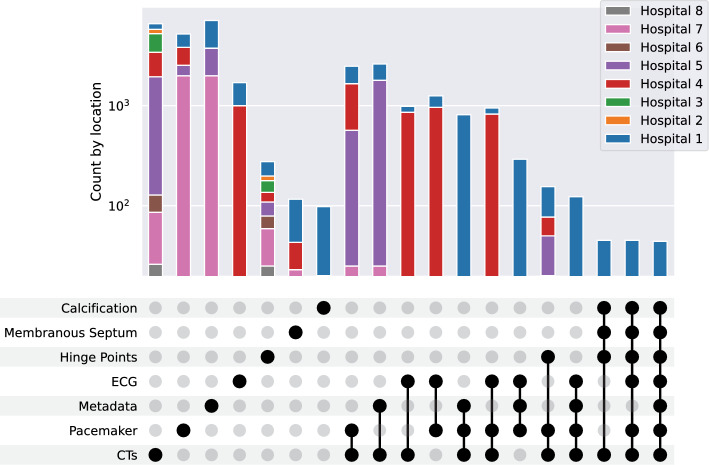
Distribution of different label data type subsets across locations on log-scale. The lower diagram displays the composition of the possible number of subset, where we restrict the subsets based on our estimation for their usefulness for independent model training. In the upper diagram the amount of samples across all locations for the particular subset of data types are visualized. Pacemaker refers to whether information (yes/no) of implantation is available

**Fig. 5:**
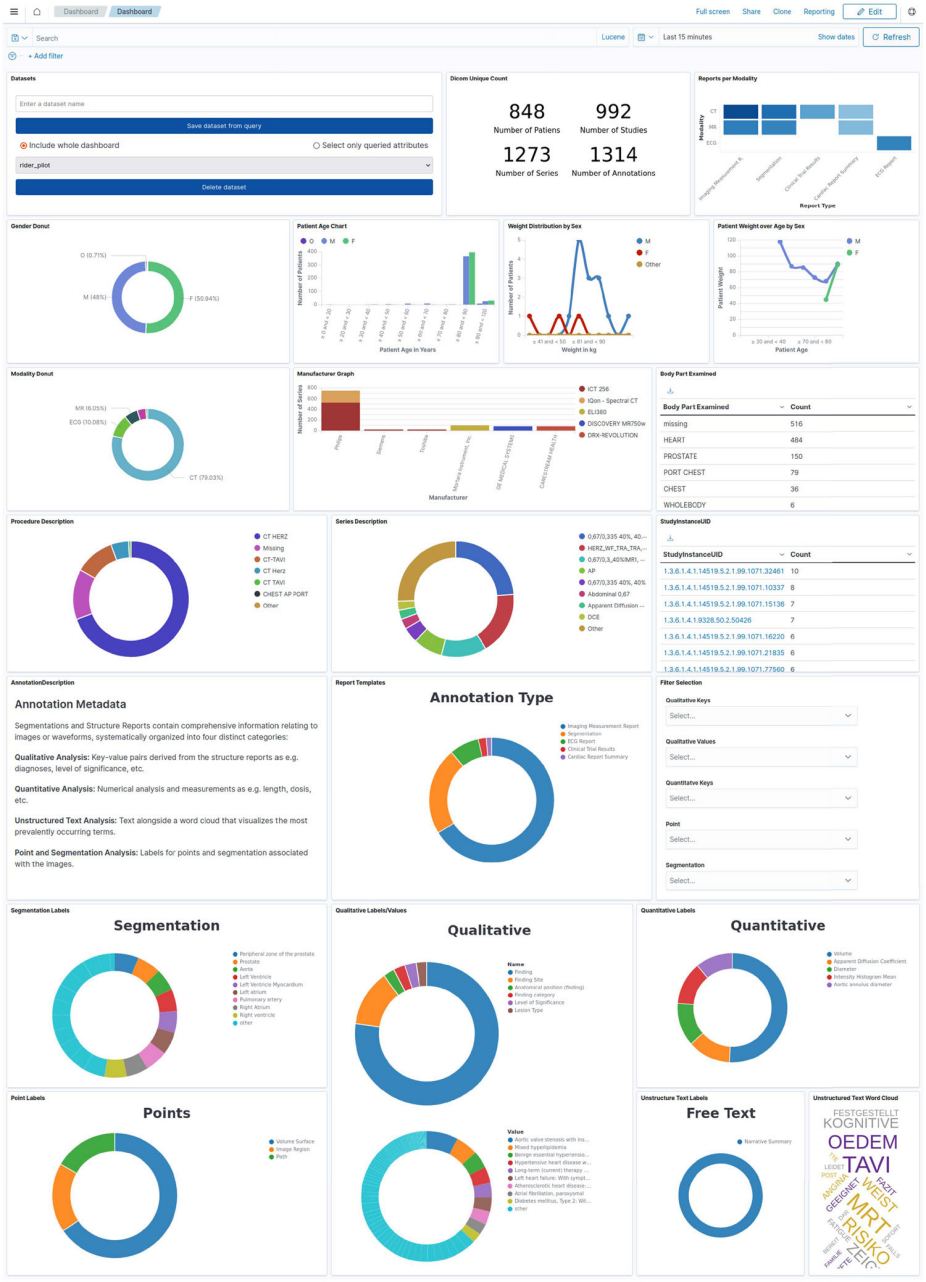
The created dashboard with the filterable annotation attributes. The type of annotation can be chosen. Segmentations can be queried on individual segment level. Since for qualitative items both name and value are a concept name, we can filter both. For numeric (e.g., measurements) as well as geometric (e.g., points) items we filter on the name. When the value is text we incorporate free text search. Some filter options concerning imaging modalities (e.g., manufacturer or body part examined) are similar to existing functionality in Kaapana, but in the backend our tool utilizes nested objects to enhance filterability with the given elements

Computed Tomography

**Description**: For each patient, potentially multiple CT scans were performed prior to TAVI, exhibiting different fields of view and contrast distributions due to varying manufacturers and scanning protocols. The intra-hospital diversity of CT scan descriptions was also large.

**Challenges**: Every patient receives multiple scans prior to TAVI that differ in protocol and number of scans taken.

**Solution**: Selecting the right series was mostly done via searching for a percentage value in the series description as this indicated the phase of the heart cycle.

Electocardiography

**Description**: ECG showed even less standardization. They varied from extensive markup language (XML)^11^ over standard communications protocol for computer assisted electrocardiography (SCP-ECG),^12^ to Hierarchical Data Format (HDF5).^13^

**Challenges**: The number of ECGs per patient varied, and matching ECGs with CT scans required identifying the temporal placement of each ECG in relation to the procedure.

**Solution**: Converting ECG data into DICOM waveform format and utilizing TID3700 ECG Report can help standardize the storage and retrieval of ECG metadata, such as ventricular heart rate or QT interval, across systems. The temporal placement of the ECG is done via the StudyDate attribute.

Quality Assurance and Billing Data

**Description**: General patient metadata was extracted from the quality assurance data for the German Institute for Quality Insurance and Transparency in Healthcare, and pacemaker information was exported from the German procedure classification used for reimbursement.

**Challenges**: Despite efforts to standardize, data varied significantly across locations and departments within hospitals as billing of different procedures is often done from different departments within the clinic. Further, mandatory fields in quality assurance questionnaires changed over the years.

**Solution**: Using TID3802 Cardiovascular Patient History can harmonize data representation across locations by allowing for the diverse storage of information, ranging from past surgical history to medical device use.

## Results

Our tool allows for the simultaneous filtering of DICOM imaging, waveform modalities, and annotations in the form of structured reports and segmentations (**R1**). All data types present in the consortium could be converted to SRs and subsequently be uploaded into the local PACS. Validation of the created SRs was done with dciodvfy from David Clunie^14^ and showed no errors. The DICOM representation ensures matching on a patient level across time points (**R2**). Filtering was performed at all locations with the same textual query (format shown in the Appendix) (**R3**). If a data type was not present at one location, the specific condition was omitted. In the case of segmentations we visualize all segment descriptions as well as the creator type (**R4**). The graphs of individual attributes update according to other queries (linked views). More filter views can be added dependent on the user requirements either with the default visualizations of Opensearch or with Vega (**R5**). A conversion of our data specifically SRs into FHIR was possible with the script provided in our Github Repo (**R6**). Querying is possible with Opensearch’s web-based query language DSL as well as with standard SQL (see Appendix). The dockerized implementation allows for a stand-alone usage as well as an integration into existing frameworks (**R7**).

SR templates exhibit a high degree of sophistication due to their intricately nested structure and the numerous attributes that can be defined [1]. To make the data filterable when uploading the SR’s data into Opensearch, the user must define which measurements, geometrical data, qualitative and text attributes shall be queryable by providing the nested path (e.g., for TID 3700 ECG Report: Cardiovascular Patient History - Social History - Tobacco Smoker). Although this might be complex at times we believe that it provides the flexibility needed for filtering a sophisticated data structure such as SRs. The definition of the path to the wanted attributes must be defined once per template, but it can be expanded or contracted at any time (**R3**). This also allows for the dynamic creation of custom templates if needed. An example for the nested, object-oriented representation of the SR template 3700 ECG report and 3708 waveform information in highdicom can be found in Fig. 2 in the Appendix.

Our chosen method was able to extract the required information from the uploaded SR templates and subsequently visualize and modify its distribution depending on the wanted cohort. The final dashboard for hospital one is shown in Fig. 5. Further resulting distribution from other locations can be found in the Appendix. The interactive graphical filtering procedure is showcased in the supplemental video.

In Heidelberg we ended up with 1273 series from 848 patients from five modalities (MRI, CT, ECG, classical X-ray (CR), digital radiography (DX)), with a variety of annotations ( 1314) comprising of segmentations (21 different labels). In total we had over 20 different types of qualitative items, 5 quantitative, 2 geometric, and 1 free text. With the help of our interface we identified 813 CT (query: “40%” in series description) and 700 ECG (query: modality is ECG) scans that can generally be used for model training. We had 78 scans with labeled hinge points and coronary arteries (HPs and CAs) (query: modality is CT AND has annotation “right coronary cusp”), 73 with membranous septum (MS) (query: modality is CT AND has annotation “membranous septum”), and 78 with annotated aortic root calcification (query: modality is CT AND has segmentation with anatomical structure “calcification”). When performing filtering at all federated locations, we obtained 6592 CT scans over all locations, 982 with CT and ECG scans (query: patient has modality CT AND ECG) across three locations, 7088 patients for which we had the implanted prosthesis information (query: series has SR with prothesis information), and 5204 patients for which we had the label of whether a pacemaker was implanted or not post-TAVI (query: series has SR with quality assurance information). The quantities within different subsets of data are shown in Fig. 4. After exporting all datasets in a unified manner to be readable by a federated learning pipeline, we can progress with training on each individual subset or the intersection of two or more. The training results are published elsewhere [25].

## Discussion

Despite their complexity, SRs provide an useful tool for unifying diagnostic reports for physicians, model training, and results when utilized appropriately (**R1**). The DICOM standard unambiguously matches patient data samples, but large cohort creation that includes patients based on specific attributes is difficult from a PACS interface (**R2**). Our solution for determining on which attributes to filter the annotations emphasizes high flexibility as it is applicable to all SR templates by concurrent conformance with other DICOM data objects such as images or waveforms. After defining the desired attributes per template the data is filterable. While writing a search query in DSL may sometimes be less intuitive compared to SQL, it offers the advantage of allowing users to copy the textual query generated through graphical filtering from one location to another, ensuring consistent results (**R3**). We believe our method is a step toward the concurrent filtering and cohort selection of DICOM data and their annotations (**R4**). Opensearch allows for custom creation of filter plots dependent on the operator’s needs, the dashboard is fully customizable (**R5**). DICOM SRs are straightforward to convert to other standards such as FHIR due to their adherence to templates (**R6**). While DICOM offers significant advantages for medical imaging studies, FHIR can be an good choice for other use cases. The platform is openly available and can be used in other projects either as a stand-alone application or as an extension to Kaapana (**R7**). The graphical filtering interface can be deployed with Docker.

With SR templates we were able to store all information in predefined templates that enable meaningful model training across locations. Still, the time needed for storing information in such an extensive standard is tedious and requires manual effort. Defining the paths to the desired attributes enabled us to filter in an intuitive way. Although this may not always preserve the original name from the SR template, it enables searching for any attribute available in any template. However, some obstacles such as inherent flattening of attributes remain due to Opensearch’s JSON based database format, which can make it challenging to retain the hierarchical structure of certain data. Additionally, while the JSON format offers high flexibility by eliminating the need to create rigid tabular indices as required in SQL, this flexibility can sometimes lead to inefficiencies in query performance. Despite these challenges, the main advantage of this approach lies in the ease of creating customized graphical filter options, enabling more intuitive and dynamic exploration of the database. This flexibility is particularly beneficial for use cases requiring quick adaptations or diverse filtering needs. Opensearch enables hierarchical filtering with nested queries, which allow for individual conditions for child elements, i.e., SRs of a CT DICOM series. Still, evaluating a single patient, for example, for the presence of both CT and ECG, required us to write textual queries. While Opensearch is built for web-based JSON queries it also allows the usage of SQL with the limitations that join queries are restricted to two indices and features like GROUP BY and ORDER BY are not supported in the results. An example of a textual query and a comparison to a SQL query of Opensearch is visualized in the Appendix.

SRs still have more inherent complexity than we have covered here, which is the main reason they are not adopted widely in clinical information systems yet, despite their obvious usefulness. The adoption of the object-oriented approach in highdicom for defining and using SR templates in Python is a step toward their more widespread usage [5, 9].

## Supplementary Information

Below is the link to the electronic supplementary material.

Supplementary file 1 (pdf 7663 KB)

Supplementary file 2 (mp4 106051 KB)

## Footnotes

1 https://gdpr.eu/.

2 https://hl7.org/fhir/.

3 https://openehr.org/.

4 https://www.medizininformatik-initiative.de/en/start.

5 https://www.kaapana.ai.

6 https://opensearch.org/.

7 https://vega.github.io/vega/.

8 https://dzhk.de/.

9 TID3802 Cardiovascular PatientHistory, TID3829 Problem Properties, TID3830 Procedure Properties, TID3831 Medical Device Use, TID 3704 Patient Characteristics for ECG, TID3702 Prior ECG Study, TID3708 ECG Waveform Information, TID3715 ECG Measurement Source, TID3713 ECG Global Measurements, TID3714 ECG Lead Measurements, TID3717 ECG Qualitative Analysis, TID3719 ECG Summary, TID3700 ECG Report, TID2002 Report Narrative Code, TID2000 Basic Diagnostic Imaging Report.

10 https://dclunie.com/dicom3tools/dciodvfy.html.

11 https://www.w3.org/TR/REC-xml/.

12 https://dicom.nema.org/medical/dicom/current/output/chtml/part17/sect_C.7.html.

13 https://docs.hdfgroup.org/hdf5/v1_14/_intro_h_d_f5.html.

14 https://dclunie.com/dicom3tools/dciodvfy.html.
