# Correction: Multi-modal dataset creation for federated learning with DICOM-structured reports

**DOI:** 10.1007/s11548-025-03409-x

**Published:** 2025-06-04

**Authors:** Malte Tölle, Lukas Burger, Halvar Kelm, Florian André, Peter Bannas, Gerhard Diller, Norbert Frey, Philipp Garthe, Stefan Groß, Anja Hennemuth, Lars Kaderali, Nina Krüger, Andreas Leha, Simon Martin, Alexander Meyer, Eike Nagel, Stefan Orwat, Clemens Scherer, Moritz Seiffert, Jan Moritz Seliger, Stefan Simm, Tim Friede, Tim Seidler, Sandy Engelhardt

**Affiliations:** 1 DZHK (German Centre for Cardiovascular Research), partner site Heidelberg/Mannheim, Heidelberg, Germany; 2 Department of Cardiology, Angiology and Pneumology, Heidelberg University Hospital, Heidelberg, Germany; 3 Informatics for Life Institute, Heidelberg, Germany; 4 DZHK (German Centre for Cardiovascular Research), partner site Hamburg/Kiel/Lübeck, Hamburg, Germany; 5 Department of Diagnostic and Interventional Radiology and Nuclear Medicine, University Medical Center Hamburg-Eppendorf, Hamburg, Germany; 6 Clinic for Cardiology III, University Hospital Münster, Münster, Germany; 7 DZHK (German Centre for Cardiovascular Research), partner site Berlin, Berlin, Germany; 8 Institute of Bioinformatics, University Medicine Greifswald, Greifswald, Germany; 9 DZHK (German Centre for Cardiovascular Research), Institute of Computer-assisted Cardiovascular Medicine, Berlin, Germany; 10 Deutsches Herzzentrum der Charité (DHZC), Institute of Computer-assisted Cardiovascular Medicine, Berlin, Germany; 11 Charité - Universitätsmedizin Berlin, Corporate Member of Freie Universität Berlin, Humboldt-Universität zu Berlin, Berlin, Germany; 12 Fraunhofer Institute for Digital Medicine MEVIS, Bremen, Germany; 13 DZHK (German Centre for Cardiovascular Research), partner site Lower Saxony, Göttingen, Germany; 14 Department of Medical Statistics, University Medical Center Göttingen, Göttingen, Germany; 15 DZHK (German Centre for Cardiovascular Research), partner site RhineMain, Frankfurt, Germany; 16 Institute for Experimental and Translational Cardiovascular Imaging, Goethe University, Frankfurt am Main, Germany; 17 DZHK (German Centre for Cardiovascular Research), partner site Munich, Munich, Germany; 18 Department of Medicine I, LMU University Hospital, LMU Munich, Munich, Germany; 19 Department of Cardiology, University Heart and Vascular Center Hamburg, University Medical Center Hamburg-Eppendorf, Hamburg, Germany; 20 Department of Cardiology, University Medicine Göttingen, Göttingen, Germany; 21 Department of Cardiology, Kerckhoff-Clinic, Campus Kerckhoff of the Justus-Liebig Universität Gießen, Gießen, Germany

**International Journal of Computer Assisted Radiology and Surgery (2025) 20:485–495** 10.1007/s11548-025-03327-y

In the original version of this article, the affiliation details for authors were incorrectly given and should have been

Malte Tölle^1,2,3^, Lukas Burger^1,2,3^, Halvar Kelm^1,2,3^, Florian André^1,2,3^, Peter Bannas^4,5^, Gerhard Diller^6^, Norbert Frey^1,2,3^, Philipp Garthe^6^, Stefan Groß^7,8^, Anja Hennemuth^5,9,10,11,12^, Lars Kaderali^7,8^, Nina Krüger^9,10,11,12^, Andreas Leha^13,14^, Simon Martin^15,16^, Alexander Meyer^9,10^, Eike Nagel^15,16^, Stefan Orwat^6^, Clemens Scherer1^7,18^, Moritz Seiffert^4,19^, Jan Moritz Seliger^4,5^, Stefan Simm^7,8^, Tim Friede^13,14^, Tim Seidler^13,20,21^, and Sandy Engelhardt^1,2,3^

^1^ DZHK (German Centre for Cardiovascular Research), partner site Heidelberg/Mannheim, Heidelberg, Germany.

^2^ Department of Cardiology, Angiology and Pneumology, Heidelberg University Hospital, Heidelberg, Germany.

^3^ Informatics for Life Institute, Heidelberg, Germany.

^4^ DZHK (German Centre for Cardiovascular Research), partner site Hamburg/Kiel/Lübeck, Hamburg, Germany.

^5^ Department of Diagnostic and Interventional Radiology and Nuclear Medicine, University Medical Center Hamburg-Eppendorf, Hamburg, Germany.

^6^ Clinic for Cardiology III, University Hospital Münster, Münster, Germany.

^7^ DZHK (German Centre for Cardiovascular Research), partner site Greifswald, Greifswald, Germany.

^8^ Institute of Bioinformatics, University Medicine Greifswald, Greifswald, Germany.

^9^ DZHK (German Centre for Cardiovascular Research), partner site Berlin, Berlin, Germany.

^10^ Deutsches Herzzentrum der Charité (DHZC), Institute of Computer-assisted Cardiovascular Medicine, Berlin, Germany.

^11^ Charité – Universitätsmedizin Berlin, Corporate Member of Freie Universität Berlin and Humboldt-Universität zu Berlin, Berlin, Germany.

^12^ Fraunhofer Institute for Digital Medicine MEVIS, Bremen, Germany.

^13^ DZHK (German Centre for Cardiovascular Research), partner site Lower Saxony, Göttingen, Germany.

^14^ Department of Medical Statistics, University Medical Center Göttingen, Göttingen, Germany.

^15^ DZHK (German Centre for Cardiovascular Research), partner site RhineMain, Frankfurt, Germany.

^16^ Institute for Experimental and Translational Cardiovascular Imaging, Goethe University, Frankfurt am Main, Germany.

^17^ DZHK (German Centre for Cardiovascular Research), partner site Munich, Munich, Germany.

^18^ Department of Medicine I, LMU University Hospital, LMU Munich, Munich, Germany.

^19^ Department of Cardiology, University Heart and Vascular Center Hamburg, University Medical Center Hamburg-Eppendorf, Hamburg, Germany.

^20^ Department of Cardiology, University Medicine Göttingen, Göttingen, Germany.

^21^ Department of Cardiology, Kerckhoff-Clinic, Campus Kerckhoff of the Justus-Liebig-Universität Gießen, Gießen, Germany.

The original article has been corrected.

