# Supplementary material for: Multi-modal dataset creation for federated learning with DICOM-structured reports
Source: Int J Comput Assist Radiol Surg. 2025 Feb 3;20(3):485–95. doi: 10.1007/s11548-025-03327-y (PMC11929732; doi:10.1007/s11548-025-03327-y)
Supplement: Supplementary file 1 — (pdf 7663 KB) [file 11548_2025_3327_MOESM1_ESM.pdf]

Appendix

Nested Queries

By default, each of the nested objects is dynamically mapped as object field type. Any object field can take an array of objects.

```
PUT meta-index/_doc/100
{ "Series_ID": "1.2.345.6.01",
  "Patient_name" : "John Doe",
  "Modality" : "MR",
  ...
  "Reports": [
    {
        "Series_ID" : "7.8.765.01",
        "ContentKeyword": "Segmentation",
        "AnatomicalStructure" : [
            "Left Atrium",
            "Aorta"
        ],
        ...
    }
  ]
}

PUT meta-index/_doc/101
{ "Series_ID": "1.2.345.6.02",
  "Patient_name" : "Jane Smith",
  "Modality" : "CT",
  ...
  "Reports": [
    {
        "Series_ID" : "8.8.765.01",
        "ContentKeyword": "Segmentation",
        "AnatomicalStructure" : [
            "Left Ventricle",
            "Right Ventricle"
        ],
        ...
    }
  ]
}

PUT meta-index/_doc/102
{ "Series_ID": "1.2.345.6.03",
  "Patient_name" : "Random Hacker",
  "Modality" : "CT",
```

```
  ...
  "Reports": [
    {
        "Series_ID" : "9.8.765.01",
        "ContentKeyword": "Segmentation",
        "AnatomicalStructure" : [
            "Left Ventricle",
            "Right Atrium"
        ],
        ...
    },
    {
        "Series_ID" : "9.8.765.02",
        "ContentKeyword": "Segmentation",
        "AnatomicalStructure" : ["Liver","Right Ventricle"],
        ...
    }
  ]
}
```

When multiple "Reports" objects are stored (eg. for 102), they are flattened, so their internal representation has an array of all values for each field:

```
{
    "Series_ID": "1.2.345.6.03"
    "Patient_name" : "Random Hacker",
    "Modality" : "CT",
    ...
    "Reports.Series_ID" : ["9.8.765.01","9.8.765.02"],
    "Reports.ContentKeyword" : [
        "Segmentation",
        "Segmentation"
    ],
    "Reports.AnatomicalStructure" : [
        "Left Ventricle",
        "Right Atrium",
        "Liver",
        "Right Ventricle"
    ],
}
```

A search for series with CT segmentation of "Left Ventricle" AND "Right Ventricle" within the same report would not work as expected and would result in an "OR" connection. This would correctly return document 101 but would also incorrectly return document 102, where the anatomical structures appear in separate reports rather than within the same one.

OpenSearch DSL-Syntax:

```
GET meta-index/_search
{
  "query": {
    "bool": {
      "must": [
        {
          "term": {
            "Modality": "CT"
          }
        },
        {
          "term": {
              "Reports.AnatomicalStructure": "Left Ventricle"
            }
        },
        {
          "term": {
            "Reports.AnatomicalStructure": "Right Ventricle"
            }
        }
      ]
    }
  }
}
```

OpenSearch SQL-API:

```
POST _plugins/_sql
{
  "query": """
    SELECT *
    FROM meta-index
    WHERE
      Modality = 'CT' AND
        (Reports.AnatomicalStructure = 'Left Ventricle' AND
        Reports.AnatomicalStructure = 'Right Ventricle');"""
}
```

Therefore, **Nested Documents** are indexed separately from the parent document, and remain linked to the parent, such that queries on nested fields can filter down to the specific objects in the array that contains all required conditions.

```
PUT meta-index
{
  "mappings" : {
    "properties": {
      "Reports": {
        "type" : "nested"
      }
    }
  }
}
```

The following nested query can be used to search for series with CT segmentation containing both "Left Ventricle" **AND** "Right Ventricle". This query correctly returns only the document with ID 101:
OpenSearch DSL-Syntax:

```
GET meta-index/_search
{
  "query": {
    "bool": {
      "must": [
        {
          "term": {
            "Modality": "CT"
          }
        },
        {
          "nested": {
            "path": "Reports",
            "query": {
              "bool": {
                "must": [
                  {
                    "match": {
                      "Reports.AnatomicalStructure":"Left V.."
                    }
                  },
                  {
                    "match": {
                      "Reports.AnatomicalStructure":"Right V."
                    }
                  }
                ]
```

```
                }
              }
            }
          }
        ]
      }
    }
}
```

OpenSearch SQL API:

```
POST _plugins/_sql
{
  "query": """
    SELECT m.Series_ID_keyword , r.Series_ID_keyword
    FROM meta-index AS m
    JOIN report-index AS r
    ON m.Series_ID_keyword = r.Ref_Series_ID_keyword
    WHERE
      m.Modality = 'CT' AND
      (r.AnatomicalStructure  = 'Left Ventricle' AND
      r.AnatomicalStructure = 'Right Ventricle');"""
}
```

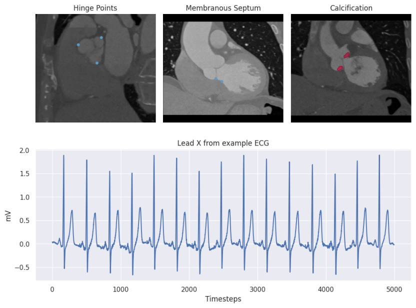

(a)

	Name	Value
CODE	Concept Name Code Sequence (Val., Scheme Designator, Meaning)	Concept Name Code Sequence (Value, Scheme Designator, Meaning)
NUM	Concept Name Code Sequence (Val., Scheme Designator, Meaning)	Measured Value Sequence<br>> Numeric Value<br>> Concept Name Code Sequence<br>(Value, Scheme Designator, Meaning)
SCOORD3D	Concept Name Code Sequence (Val., Scheme Designator, Meaning)	Graphic Type<br>Graphic Data<br>Frame of Reference UID
TEXT	Concept Name Code Sequence (Val., Scheme Designator, Meaning)	Text

(b)

Fig. 1: (a) Example computed tomography and electrocardiography scan with corresponding labels. From left to right: hinge points of the cusps of the aortic valve, start and end point of the membranous septum, and aortic root calcification. The CT scans also vary greatly in terms of contrast. (b) While SRs exhibit highly nested structures, at their core they are comprised of simple data types that are consistent across templates.

Table 1: Parameters per modality queryable in the filter tool and available for model training.

**Modality**	**Parameter**
Quality Assurance	ACE Inhibitor, Age, Aldosterone Antagonist, Angina Pectoris, ASA, Bacteremia, Beta Blocker, Bleeding, Bronchopulmonary Infection, COPD, Complication, Coronary Angiography Report, Creatinine, Decompensation, Degree of Disability, Diabetes, Diuretics, Dissection, Florid Endocarditis Infection, Heart Rhythm, Height, Hematoma, Hemiblock, Hepatitis Infection, HIV Infection, Infarct, Inotrope, Ischemia, LVEF, Lung, Lower Extremity Wound Infection, Main Trunk Stenosis, Malignom, Mediastinitis Infection, Medicinal Therapy Heart Failure, Negative T Wave, Neurology, Nitrate, No Infection, NYHAE, Otolaryngological Infection, Other Infection, Other Therapy Heart Failure, Other Wound Infection, PCI, Peritonitis Infection, Previous Operations, Pulmonary Hypertension, Reason Age, Reason for Rethoracotomy, Reason Frailty, Reason High Risk, Reason Porcellain Aorta, Reason Secondary Disease, Reanimation, Renal Replacement Therapy, Revascularisation Indicated, Sepsis Infection, Sex, Troponin, Urinary Tract Infection, Vascular Complication, Ventilation, Venous Catheter Infection, Weight
Electocardiography	Atrial Fibrillation, Atrial Rate, AV Block, AVK, Bifascicular Block, Bradycardia, Left Bundle Branch Block, Left Ventricular Hypertrophy, Myocardial Infarction, PAxis, POffset, POnset, PRInterval, QOffset, QOnset, QRSCount, QRSDuration, QTCorrected, QTInterval, QT Prolongation, QTcFrederica, RAxis, Right Bundle Branch Block, RSR Pattern, ST Depression, ST Elevation, TAxis, Tachycardia, TOffset, VentricularRate
Computed Tomography	Aortic Annulus Plane measurements, Calcification, Left Ventricular Outflow Tract measurements, Location of Membranous Septum, Sinotubular Junction measurements, Sinus of Valsalva measurements
Prosthesis	Manufacturer, Model, Size

Table TID 3700. ECG Report

	NL	Rel with Parent	VT	Concept Name	VM	Req Type
1			CONTAINER	EV (28010-7, LN, "ECG Report")	1	M
2	>	HAS CONCEPT MOD	CODE	EV (121058, DCM, "Procedure reported")	1	U
3	>	HAS CONCEPT MOD	INCLUDE	DTID 1204 "Language of Content Item and Descendants"	1	M
4	>	CONTAINS	INCLUDE	DTID 1002 "Observer Context"	1-n	M
5	>	CONTAINS	CONTAINER	EV (18785-6, LN, "Indications for Procedure")	1	U
6	>>	CONTAINS	CODE	EV (121071, DCM, "Finding")	1-n	U
7	>>	CONTAINS	TEXT	EV (121071, DCM, "Finding")	1	U
8	>	CONTAINS	INCLUDE	DTID 3802 "Cardiovascular Patient History"	1	U
9	>	CONTAINS	INCLUDE	DTID 3704 "Patient Characteristics for ECG"	1	U
10	>	CONTAINS	INCLUDE	DTID 3702 "Prior ECG Study"	1	U
11	>	CONTAINS	INCLUDE	DTID 3708 "ECG Waveform Information"	1	M
12	>	CONTAINS	CONTAINER	EV (122144, DCM, "Quantitative Analysis")	1	M
13	>>	CONTAINS	INCLUDE	DTID 3713 "ECG Global Measurements"	1	U
14	>>	CONTAINS	INCLUDE	DTID 3714 "ECG Lead Measurements"	1-n	U
15	>	CONTAINS	INCLUDE	DTID 3717 "ECG Qualitative Analysis"	1	U
16	>	CONTAINS	INCLUDE	DTID 3719 "Summary, ECG"	1	U

(a)

```python
class ECGReport(Template):
    """:dcm:`TID 3700 <part16/chapter_A.html#sect_TID_3700>`
    ECG Report
    """
    def __init__(
        self,
        language_of_content_item_and_descendants: LanguageOfContentItemAndDescendants,
        observer_contexts: Sequence[ObserverContext],
        ecg_waveform_information: ECGWaveFormInformation,
        ...
    ) -> None:
        item = ContainerContentItem(
            name=codes.LN.ECGReport,
            template_id='3700'
        )
        content = ContentSequence()
        content.extend(language_of_content_item_and_descendants)
        for observer_context in observer_contexts:
            content.extend(observer_context)
        content.extend(ecg_waveform_information)
        ...
        self.append(item)
```

(b)

```python
class ECGWaveFormInformation(Template):
    """:dcm:`TID 3708 <part16/sect_TID_3708.html>`
    ECG Waveform Information
    """
    def __init__(
        self,
        procedure_datetime: Union[str, datetime.datetime, DT],
        source_of_measurement: Optional[WaveformContentItem] = None,
        lead_system: Optional[Union[CodedConcept, Code]] = None,
        acquisition_device_type: Optional[str] = None,
        ...
    ) -> None:
        item = ContainerContentItem(
            name=codes.LN.CurrentProcedureDescriptions,
            template_id='3708'
        )
        content = ContentSequence()
        procedure_datetime_item = DateTimeContentItem(
            name=codes.DCM.ProcedureDatetime,
            value=procedure_datetime,
            relationship_type=RelationshipTypeValues.CONTAINS
        )
        content.append(procedure_datetime_item)
        if source_of_measurement is not None:
            content.append(source_of_measurement)
        if lead_system is not None:
            lead_system_item = CodeContentItem(
                name=CodedConcept(
                    value='10:11345',
                    meaning='Lead System',
                    scheme_designator='MDC'
                ),
                value=lead_system,
                relationship_type=RelationshipTypeValues.CONTAINS
            )
            content.append(lead_system_item)
        if acquisition_device_type is not None:
            acquisition_device_type_item = TextContentItem(
                name=codes.DCM.AcquisitionDeviceType,
                value=acquisition_device_type,
                relationship_type=RelationshipTypeValues.CONTAINS
            )
            content.append(acquisition_device_type_item)
```

(c)

Fig. 2: Schematic overview over structured reports. (a) Tabular representation of TID 3700 ECGReport from the official DICOM website[15]. (b) Object oriented definition of the template in `highdicom`. (c) Also sub-templates can be stored in Python-classes, which makes them reusable across all templates.

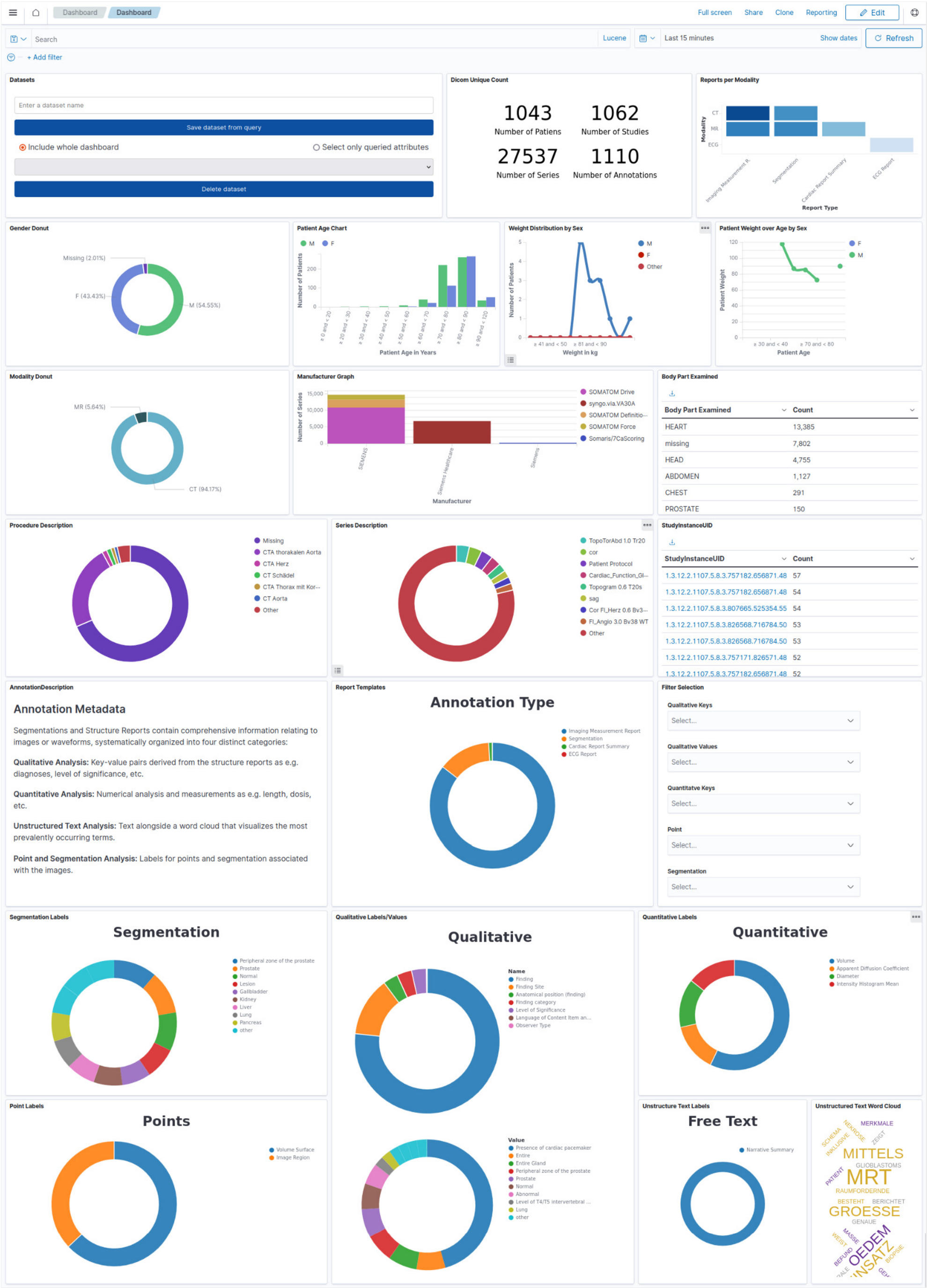

Fig. 3: The created, unfiltered dashboard with the queryable annotation attributes in hospital two.

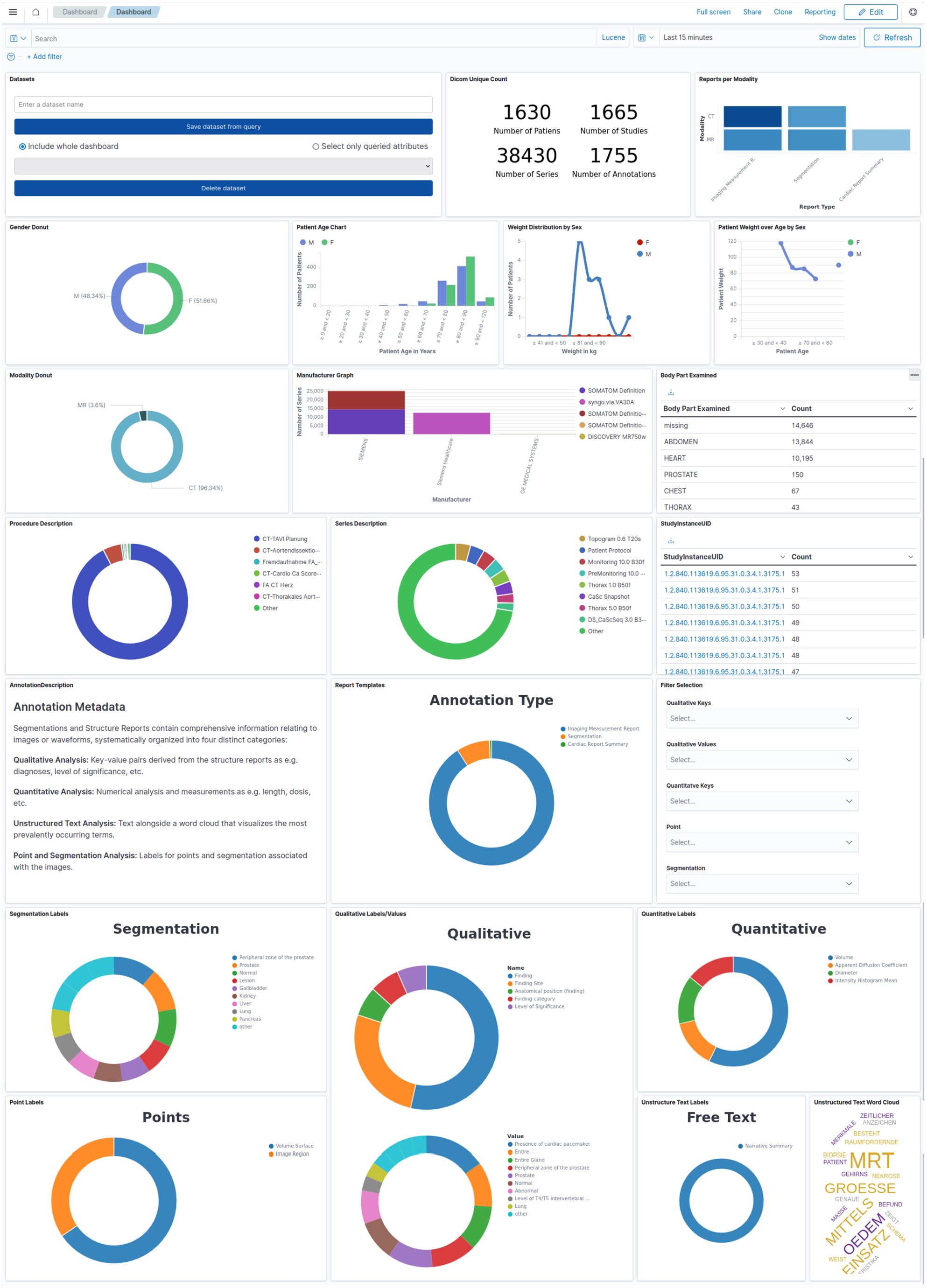

Fig. 4: The created, unfiltered dashboard with the queryable annotation attributes in hospital three.
